# Exploring lymphocyte subsets in COVID-19 patients: insights from a tertiary academic medical center with a high proportion of patients on immunosuppression

**DOI:** 10.3389/fimmu.2024.1436637

**Published:** 2024-12-03

**Authors:** Katrijn Daenen, Samantha van Hooijdonk, Kirby Tong-Minh, Willem A. Dik, Petrus M. van Hagen, Jilske A. Huijben, Diederik Gommers, Eric C. M. van Gorp, Henrik Endeman, Virgil A. S. H. Dalm

**Affiliations:** ^1^ Department of Intensive Care, Erasmus University Medical Center, Rotterdam, Netherlands; ^2^ Department of Viroscience, Erasmus University Medical Center, Rotterdam, Netherlands; ^3^ Department of Immunology, Erasmus University Medical Center, Rotterdam, Netherlands; ^4^ Laboratory Medical Immunology, Department of Immunology, Erasmus University Medical Center, Rotterdam, Netherlands; ^5^ Department of Internal Medicine, Division of Allergy & Clinical Immunology, Erasmus University Medical Center, Rotterdam, Netherlands; ^6^ Department of Internal Medicine, Erasmus University Medical Center, Rotterdam, Netherlands

**Keywords:** COVID-19, lymphocyte subsets, infectious diseases, severity, prediction, immunosuppression

## Abstract

**Introduction:**

Severe COVID-19 is associated with reduced absolute lymphocyte counts, suggesting that lymphocyte subsets may serve as predictors of clinical outcomes in affected patients. Early identification of patients at risk for severe disease is crucial for optimizing care, accurately informing patients and their families, guiding therapeutic interventions, and improving patient flow in the ED. Given that immunosuppressive drugs significantly impact lymphocyte profiles, we aimed to determine the association between prior use of immunosuppressive drugs, lymphocyte subsets, and COVID-19 severity in our population with a high prevalence of immunosuppression.

**Methods:**

In 2021, suspected COVID-19 patients were included in the ED. Lymphocyte subsets were determined in peripheral blood within 24 hours after presentation and comparative analyses was performed between SARS-CoV-2 negative and positive patients, mild versus severe disease and patients with and without prior immunosuppressive drug use. Mild cases were patients discharged home or admitted to a general ward, severe cases were patients with COVID-19-related mortality or necessitating ICU admission. Logistic regression analysis was performed to assess the association between lymphocyte subsets and COVID-19 severity, and between prior immunosuppressive drug use and COVID-19 severity.

**Results:**

Twenty-five SARS-CoV-2 negative and 77 SARS-CoV-2 positive patients were included, whereof 57 (74%) had mild and 20 (26%) severe COVID-19. No significant differences were observed in the absolute counts of CD3+, CD4+, and CD8+ T-lymphocytes, B-lymphocytes, and NK-cells between SARS-CoV-2 negative and positive patients or between mild and severe cases. The 36 patients with prior use of immunosuppressive drugs had significantly lower CD4+ T-lymphocytes (p<0.01). Prior use of immunosuppressive drugs was not associated with COVID-19 severity (adjusted OR 1.074, 0.355-3.194).

**Conclusion:**

Lymphocyte subsets were not significantly different between SARS-CoV-2 negative and positive patients and between mild versus severe cases. Neither lymphocyte subsets nor prior immunosuppressive drug use were associated with COVID-19 severity.

## 1 Introduction

The emergence of the severe acute respiratory syndrome coronavirus 2 (SARS-CoV-2) in Wuhan, China, causing the Coronavirus disease 2019 (COVID-19), led to a global pandemic. Initially, COVID-19 was associated with a high risk of morbidity and mortality (1). Although the risk of severe disease has decreased in the general population due to acquired immunity through natural infection or vaccination, specific patient groups remain vulnerable, including immunocompromised patients (2–5). Upon hospital presentation, COVID-19 patients show a broad clinical spectrum, ranging from mild respiratory symptoms to life-threatening acute respiratory distress syndrome (ARDS) (6–9). Anticipating the course of their disease in an early state remains challenging and requires further understanding.

Early identification of patients at risk for severe disease is crucial for optimizing care, accurately informing patients and their families, guiding therapeutic interventions, and improving patient flow in the ED (10). Furthermore, in times of limited hospital admission capacity it is imperative to identify patients at low risk of severe outcomes, so home-based monitoring or treatment can be considered (11).

One approach that might identify patients at risk for severe outcome is lymphocyte subset analysis (12–15). Lymphocytes play a pivotal role in coordinating innate and adaptive immune response, and serve as key players in the recognition and elimination of viral pathogens, including SARS-CoV-2. Patients with severe COVID-19 often show reduced absolute lymphocyte counts, particularly CD4+ T- lymphocytes and CD8+ T-lymphocytes (16, 17). Therefore, exploring the association between lymphocyte subsets and severe outcomes in patients presenting to the ED holds interest.

Although the exact impact of the use of immunosuppressive drugs on outcome in COVID-19 remains incompletely understood, it is generally found that immunosuppressed individuals have an increased risk of severe outcome (2, 4, 18, 19). The increased susceptibility to severe COVID-19 might be attributable to an absolute decrease in lymphocyte subsets or to other factors such as functional impairment of immune cells, disturbed response to vaccination, or specific patient characteristics like comorbidities (5, 20). When treating patients with immunosuppressive drugs, a delicate balance needs to be found between suppressing the immune system and maintaining its ability to defend against pathogens. Early testing of lymphocyte subsets might provide diagnostic and therapeutic guidance in these patients.

In this study, conducted at our tertiary academic medical center with a high prevalence of immunosuppressive drug use, we hypothesize that differences in lymphocyte subsets are associated with severe or fatal COVID-19 disease. Our aims are to compare lymphocyte subsets between SARS-CoV-2 negative and positive patients, as well as in mild versus severe COVID-19 disease, and to evaluate the association between lymphocyte subsets and the severity of COVID-19. Additionally, we study the association between prior immunosuppressive drug use on lymphocyte subsets and COVID-19 severity.

## 2 Methods

### 2.1 Study participants and design

This single-center cohort study was conducted at the Erasmus MC in Rotterdam, a tertiary academic medical center in the Netherlands. Patients were enrolled between March 10, 2021 and June 4, 2021 at the ED. Patients were eligible for inclusion if they presented at the ED with suspected COVID-19, were at least 18 years of age, and provided informed consent. A suspected SARS-CoV-2 infection was defined as a patient having complaints that could fit the diagnosis COVID-19 including: dry cough, fever, headache, diarrhea, dyspnea, rhinitis and/or lack of taste or scent. SARS-CoV-2 positivity was diagnosed by a positive nasopharyngeal swab for SARS-CoV-2 detected through reverse- transcriptase polymerase chain reaction (RT-PCR). Exclusion criteria for participation were insufficient knowledge of the Dutch language and disabilities unrelated to COVID-19. Follow-up was defined as date of ED presentation until 30 days after discharge. Patients directly transferred from the ED to the ICU could not be included due to logistical reasons. If providing informed consent was not possible, e.g. in case of sedation for invasive mechanical ventilation and/or lack of consciousness, informed consent could be obtained from a legal representative. Withdrawal of participation was possible at all times.

This study was approved by the local Medical Ethics Review Committee of the Erasmus University Medical Center (Erasmus MC) under protocol number MEC- 2020-0337 and conducted according to the principles of the World Medical Association Declaration of Helsinki. All participants or their legal representatives provided written informed consent before enrollment.

### 2.2 Data collection

Peripheral blood samples were drawn at the ED within 24 hours after presentation from all patients in three Na-heparin tubes and one serum separating tube. To determine lymphocyte subsets, flow cytometric analysis was performed on the collected peripheral blood samples. This analysis was conducted in an ISO15189 certified laboratory, utilizing a FACS CantoII instrument manufactured by Becton Dickinson (14). Lymphocyte subsets, including CD3+ T-lymphocytes, CD4+ T-lymphocytes, CD8+ T-lymphocytes, B-lymphocytes, and NK-cells were defined as follows: CD3+ T-lymphocytes as CD45+CD3+CD4-CD8-, CD4 T-lymphocytes as CD45+CD3+CD4+CD8-, CD8 T-lymphocytes as CD45+CD3+CD8+CD4-, B-lymphocytes as CD45+CD3-CD16/56-CD19+, and NK-cells as CD45+CD3-CD19-CD16/56+.

Demographic data, pre-existing comorbidities, medication use, laboratory parameters, vital parameters, use of respiratory support, treatment and outcome data were obtained from electronic medical records. Information on COVID-19 complaints and COVID-19 vaccination status was collected at inclusion. The following laboratory parameters were included in this study: C-reactive protein (CRP), procalcitonin, ferritin, lactate dehydrogenase (LDH), white blood cell count, red blood cell count, platelets and the lymphocyte subsets as described. Respiratory support was defined as oxygen therapy by means of a nasal cannula, non- rebreathing mask, high-flow oxygen therapy or invasive mechanical ventilation. Immunosuppressive drug use was defined as the use of systemic corticosteroids >7,5 mg prednisone per day or equivalent, TNF-a inhibitors, mycophenolate mofetil, calcineurin blockers, azathioprine, methotrexate, hydroxychloroquine, interleukin antagonists or any other type of immunosuppressive drug use.

### 2.3 Study outcome

The study population was divided into two groups: SARS-CoV-2 negative and SARS-CoV-2 positive patients, confirmed by RT-PCR. Within the group of SARS-CoV-2 positive patients, the primary outcome was categorization into either mild or severe COVID-19 disease. Mild COVID-19 was defined as admission to the general ward or no hospitalization requirement, while severe COVID-19 was defined as requiring ICU admission or COVID-19 related mortality, as assessed by the treating physician’s clinical judgment.

### 2.4 Statistical analysis

Baseline characteristics were described as frequencies with percentages for categorical variables and, continuous variables were described by using either median and interquartile ranges (IQR) or mean and standard deviations (SD). White blood cell counts and all lymphocyte subsets were compared between SARS-CoV-2 negative and positive patients, between mild and severe COVID-19 cases and between SARS-CoV-2 positive patients with and without prior immunosuppressive drug use by using the Pearson’s Chi-square test or Fisher’s exact test for categorical data, and the Student’s t-test and Mann-Whitney U test (non-normal distributions) for continuous variables. Standardized mean differences (SMD) with confidence intervals were calculated to quantify the magnitude of differences between groups. Furthermore, we calculated the ORs of the lymphocyte subsets for mild versus severe COVID-19 using multivariate logistic regression analysis adjusting for age, sex and BMI. Additionally, we examined the association between prior immunosuppressive drug use and COVID-19 severity, adjusting for age, sex, and white blood cell counts. Missing data were imputed using multiple imputation when more than 2.5% of a variable was missing. The imputation model included all available clinical characteristics, and five imputed datasets were generated for statistical analysisA p-value of less than 0.05 was considered statistically significant. For statistical analysis IBM SPSS Statistics version 25 was used.

## 3 Results

**Figure 1:**
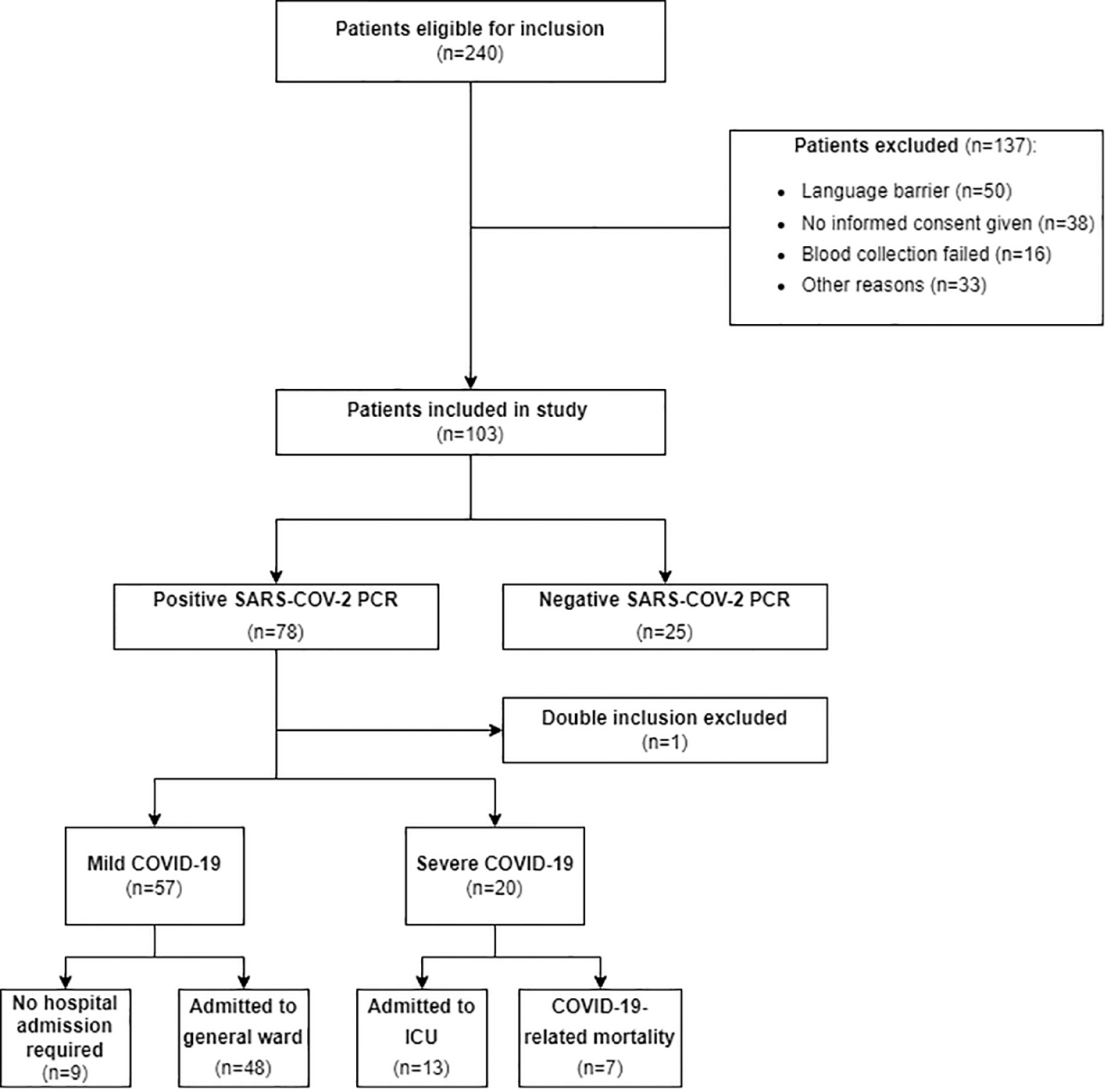
Flow diagram of patient selection. This figure shows the flow diagram detailing the patient selection process for our study, indicating the number of patients included and excluded, along with the reasons for exclusion. Mild COVID-19 was defined as patients admitted to a general ward or discharged home and severe COVID-19 was defined as patients requiring ICU admission or experiencing COVID-19-related mortality. SARS-CoV-2, Severe Acute Respiratory Syndrome Coronavirus 2; PCR, transcriptase polymerase chain reaction; ICU, Intensive Care Unit.

From March 10, 2021, to June 4, 2021, a total of 240 suspected COVID-19 patients were eligible for inclusion and 103 patients were included in the study. Reasons for exclusion included a language barrier, blood collection failure, lack of informed consent, and other miscellaneous reasons. Within the ‘no informed consent’ group, 17 patients were excluded because they were directly admitted to the ICU from the ED, which prevented obtaining informed consent. One patient was included twice, so only data from the first inclusion was considered for analysis. Of the included patients, 25 patients tested negative and 77 patients tested positive for SARS-CoV-2. Fifty-seven patients were classified as having mild COVID-19, and 20 patients were classified as having severe COVID-19 (**Figure 1**).

### 3.1 Clinical characteristics of SARS-CoV-2 positive patients

The patient characteristics at baseline of the SARS-CoV-2 positive patients, categorized into mild and severe COVID-19, are presented in **Table 1**. No differences were found in age between the mild COVID-19 patients [mean 59 years (IQR 46.0-69.0)] and severe COVID-19 patients [mean 57 years (IQR 45-71)]. In both groups the majority of patients were male and BMI was significantly higher in the severe COVID-19 group (p<0.01). From all the reported comorbidities, cardiovascular comorbidities were most common in both groups. Pulmonary disease was the only comorbidity with a significantly higher prevalence in the severe COVID-19 group (p<0.01). The interval between initial onset of clinical symptoms and presentation at the ED ranged from 0 to 21 days, with a median of 8 days (IQR 5.0-10.5). There was no significant difference in initial symptoms between the mild COVID-19 group and the severe COVID-19 group (p=0.571). The only vital parameter that showed a statistically significant difference between patients with mild and severe COVID-19 was the respiratory rate (p<0.005). Severe COVID-19 patients were more often in need of respiratory support at presentation at the ED (p<0.01) and had a significantly longer hospital stay (p<0.01). Of the 20 patients with severe COVID-19, the median time to ICU admission following ED presentation was 3 days (IQR: 1–8 days). COVID-19-related mortality was observed in 7 of these patients.

**Table 1:** Baseline patient characteristics mild and severe COVID-19.

		TOTAL (N= 77)	MILD COVID-19 (N=57)	SEVERE COVID-19 (N=20)	P-VALUE
AGE	Years, median (IQR)	58 (46-69)	59 (46-69)	57 (45-71)	0.71
SEX	Men, N (%)	49 (64%)	38 (67%)	11 (55%)	0.35
BMI	Mean (SD)	30.19 (6.69)	28.82 (5.33)	34.09 (8.60)	<0.01
COMORBIDITIES	Central nervous system disease, n (%)	8 (11%)	5 (9%)	3 (15%)	0.42
	Pulmonary disease, n (%)	20 (260%)	10 (18%)	10 (50%)	<0.01
	Diabetes mellitus, n (%)	14 (18%)	11 (19%)	3 (15%)	1.00
	Renal disease, n (%)	11 (14%)	8 (14%)	3 (15%)	1.00
	Liver disease, n (%)	7 (9%)	7 (12%)	0	0.18
	Active malignancy, n (%)	13 (17%)	9 (16%)	4 (20%)	0.73
	Immunodeficiency, n (%)	7 (9%)	4 (7%)	3 (15%)	0.37
	Auto-immune disease, n (%)	11 (14%)	8 (14%)	3 (15%)	1.00
	Organ transplant, n (%)	10 (13%)	8 (14%)	2 (10%)	1.00
IMMUNOSUPPRESSIVE DRUG USE	Immunosuppressive use, n (%)	36 (47%)	26 (46%)	10 (50%)	0.74
	Duration (months), median (IQR)	10.5 (0.0-60.8)	14.0 (0.0-60.3)	8.0 (0.0-71.5)	0.94
VACCINATION STATUS	0 vaccines, n (%)	66 (86%)	49 (86%)	17 (85%)	1.00
	1 vaccines, n (%)	9 (12%)	7 (12%)	2 (10%)	1.00
	2 vaccines, n (%)	2 (3%)	1 (2%)	1 (5%)	0.46
COVID-19 SYMPTOMS	Time onset symptoms to admission, median (IQR)	8.00 (5.00-10.50)	9.00 (5.00-11.00)	7.00 (5.25-10.00)	0.57
	Fever, n (%)	64 (83%)	46 (81%)	18 (90%)	0.50
	Cough, n (%)	67 (87%)	49 (86%)	18 (90%)	1.00
	Dyspnea, n (%)	58 (75%)	40 (70%)	18 (90%)	0.13
	Diarrhea, n (%)	37 (48%)	28 (49%)	9 (45%)	0.75
	Headache, n (%)	48 (62%)	36 (63%)	12 (60%)	0.80
	Sore throat, n (%)	26 (34%)	19 (33%)	7 (35%)	0.89
	Lack of taste or scent, n (%)	36 (47%)	26 (46%)	10 (50%)	0.74
	Rhinitis, n (%)	30 (39%)	19 (33%)	11 (55%)	0.087
VITAL PARAMETERS	Temperature (Celsius), mean (SD)	37.6 (1.0)	37.5 (1.0)	38.0. (1.0)	0.074
	Respiratory rate (/min), mean (SD)	19 (5)	18 (5)	22 (5)	<0.01
	SpO2 (%), mean (SD)	95 (2)	96 (3)	95 (1)	0.69
	Heart rate (BPM), mean (SD)	84 (14)	86 (14)	83 (14)	0.49
	Systolic blood pressure (mmHg), mean (SD)	132 (17)	130 (17)	133 (17)	0.46
	Diastolic blood pressure (mmHg), mean (SD)	78 (11)	77 (12)	79 (10)	0.45
RESPIRATORY SUPPORT	Any type of respiratory support at admission, n (%)	49 (64%)	31 (55%)	18 (90%)	<0.01
	Nasal cannula, n (%)	42 (55%)	30 (53%)	12 (60%)	0.57
	Non-rebreathing mask, n (%)	6 (8%)	1 (2%)	5 (25%)	<0.01
	OptiFlow, n (%)	1 (1%)	0	1 (5%)	0.26
LABORATORY PARAMETERS	C-reactive protein (mg/L), median (IQR)	63 (25-120)	56 (21-116)	83 (60-166)	<0.05
	Procalcitonin (ng/ml), median (IQR)*	0.13 (0.07-0.35)	0.13 (0.07-0.31)	0.17 (0.10-0.40)	0.44
	Ferritin (µg/L), median (IQR)*	905 (462-1563)	1017 (515-1659)	722 (403-1136)	0.41
	Lactate Dehydrogenase (U/L), median (IQR)*	340 (275-420)	322 (256-394)	383 (339.-531)	0.01
	Red blood cell count (x10^12^/L), median (IQR)	4.50 (4.03-5.08)	4.50 (4.03-5.16)	4.50 (4.05-4.72)	0.62
	Platelets (x10^9^/l), median (IQR)	196 (156-267)	205 (127-280)	189 (146-241)	0.24
LENGTH OF HOSPITAL STAY	Days, median (IQR)	6 (2-14)	4 (2-8)	22 (14-32)	<0.01
LENGTH OF ICU STAY	Days, median (IQR)	NA	NA	14 (6-24)	NA

This table shows the baseline characteristics of mild versus severe COVID-19 patients and the total patient population. Mild COVID-19 was defined as patients admitted to a general ward or discharged home and severe COVID-19 was defined as patients requiring ICU admission or experiencing COVID-19-related mortality. The vital, respiratory and laboratory parameters were assessed at presentation to the ED. *Missing data were observed in 15.7% of patients for procalcitonin, 14.7% for ferritin and 2.9% for LDH.

BMI, body mass index; BPM, beats per minute; IQR, interquartile range; SD, standard deviation.

Forty-seven percent (N=36) of all SARS-CoV-2 positive patients were using any immunosuppressive drugs prior to infection. In the severe COVID-19 group this was 50.0% (N=10), and in the mild COVID-19 group this was 45.6% (N=26). Among SARS-CoV-2 positive patients discharged home from the ED, only 1 patient (11.1%) was on immunosuppressive drugs prior to presentation, whereas there were 25 patients (52.1%) in the hospitalized group that were on immunosuppressive drugs. In total, eight distinct types of immunosuppressive drugs were used, and the remaining medications were grouped in the ‘others’ category. Corticosteroids were the most frequently prescribed immunosuppressive drugs (n=36). Calcineurin inhibitors were used by twelve patients, while eleven patients used Mycophenolate mofetil. Twenty-seven patients were using more than one type of immunosuppressive drugs at the same time. Comprehensive information on the specific types of immunosuppressive drugs is shown in **Table 2**. In SARS-CoV-2 positive patients, nine patients (11.7%) had received one vaccination before they tested positive for SARS-CoV-2. Two patients got infected with SARS-CoV-2 despite being fully vaccinated with the Moderna vaccine (2 vaccinations), of which one had to be admitted to the ICU due to respiratory failure. Both patients had a history of solid organ transplant, for which they used immunosuppressive drugs. Ten of the patients that were on immunosuppressive drugs prior to presentation were vaccinated (27.8%), of whom five had received one vaccination before they tested positive for SARS-CoV-2 and five were fully vaccinated.

**Table 2:** Types of immunosuppressive drug use.

Immunosuppressive drugs	Number of patients (N=36)
Corticosteroids	36 (100%)
Mycofenolaatmofetil	11 (30.6%)
Calcineurin inhibitors	12 (33.3%)
Azathioprine	2 (5.6%)
Methotrexate	3 (8.3%)
Hydroxychloroquine	2 (5.6%)
TNF-a blockers	2 (5.6%)
Interleukin antagonist	4 (11.1%)
Others	5 (13.9%)

This table provides an overview of the specific types of immunosuppressive drugs used by patients prior to COVID-19 diagnosis. Corticosteroids were defined as the use of systemic corticosteroids >7,5mg prednisone equivalent per day. In the ‘others’ category, drugs included mesalazine (n=1), monoclonal antibodies (n=1), immunoglobulins (n=2), and bosutinib (n=1). Patients could use more than one type of immunosuppressive drugs simultaneously.

TNF-a, tumor necrosis factor alpha.

### 3.2 Lymphocyte subsets in SARS-CoV-2 negative and SARS-CoV-2 positive patients

Lymphocyte subsets were compared between SARS-CoV-2 negative and SARS-CoV-2 positive patients (**Table 3**). The absolute count of CD3+ T-lymphocytes was significantly lower in the SARS-CoV-2 positive group (median 0.427, IQR 0.284-0.589 versus median 0.689, IQR 0.321-1.066, p<0.05). No significant differences were found in white blood cell count between SARS-CoV-2 negative patients (median 6.10, IQR 3.10-11.10) and SARS-CoV-2 positive patients (median 4.60, IQR 3.30-6.55)0, nor did absolute CD4+ T-lymphocyte levels differ significantly between SARS-CoV-2 negative patients (median 0.389, IQR 0.243-0.617) and SARS-CoV-2 positive patients (median 0.286, IQR 0.183-0.433).

**Table 3:** Lymphocyte subsets at admission in SARS-CoV-2 negative and SARS-CoV-2 positive patients.

	NORMAL RANGE	TOTAL (N=102)	SARS-COV-2 NEGATIVE (N=25)	SARS-COV-2 POSITIVE (N=77)	SMD (95% CI)	P-VALUE
WHITE BLOOD CELL COUNT (10^9^/L), MEDIAN (IQR)	4-10	5.20 (3.30-7.03)	6.10 (3.10-11.10)	4.60 (3.30-6.55)	-0.593 (-0.686 - -0.500)	0.051
CD3+ T-CELLS (10^9^/L), MEDIAN (IQR)	0.7-2.1	0.459 (0.288-0.691)*	0.689 (0.321-1.066)	0.427 (0.284-0.589)	-0.682 (-0.775 - -0.589)	<0.05
CD4+ T-CELLS (10^9^/L), MEDIAN (IQR)	0.3-1.4	0.308 (0.196-0.461)*	0.389 (0.243-0.617)	0.286 (0.183-0.433)	-0.460 (-0.553 - -0.367)	0.062
CD8+ T-CELLS (10^9^/L), MEDIAN (IQR)	0.2-0.9	0.145 (0.076-0.278)*	0.163 (0.076-0.398)	0.131 (0.075-0.254)	-0.603 (-0.696 - -0.510)	0.13
B-CELLS (10^9^/L), MEDIAN (IQR)	0.1-0.5	0.099 (0.047-0.191)*	0.111 (0.043-0.225)	0.099 (0.050-0.193)	-0.222 (-0.315 - -0.129)	0.90
NK-CELLS (10^9^/L), MEDIAN (IQR)	0.09-0.6	0.106 (0.064-0.159)*	0.082 (0.038-0.286)	0.109 (0.075-0.156)	-0.320 (-0.413 - -0.227)	0.39

This table shows the baseline values of lymphocyte subsets of SARS-CoV-2 negative versus positive patients and the values of the total population determined within 24 hours from ED presentation. SARS-CoV-2 infection was diagnosed by a positive nasopharyngeal swab detected through RT-PCR.

*Missing data were observed in 2.9% of patients for CD3+ T-cells, CD4+ T-cells, CD8+ T-cells, B-lymphocytes, and NK-cells IQR = interquartile range. SMD, standardized mean difference; CI, confidence interval.

### 3.3 Lymphocyte subsets and laboratory parameters in severe and mild COVID-19 patients

The analysis of absolute counts of lymphocyte subsets in severe versus mild COVID-19 patients is shown in **Table 4**, and no significant differences were observed. In the overall cohort of SARS-CoV-2 positive patients, the medians of CD3+, CD4+, and CD8+ lymphocyte counts were below the normal range, while the medians of B-lymphocytes and NK cell counts were within the normal range. In univariate logistic regression analysis, none of the lymphocyte subsets were significantly associated with severe COVID-19 (**Supplementary Table S1**). Only levels of CRP and LDH at baseline were significantly higher in the severe COVID-19 group compared to the mild COVID-19 (p ≤ 0.01).

**Table 4:** Lymphocyte subsets at admission in mild COVID-19 versus severe COVID-19.

	NORMAL RANGE	TOTAL (N= 77)	MILD COVID-19 (N=57)	SEVERE COVID-19 (N=20)	SMD (95% CI)	P-VALUE
WHITE BLOOD CELL COUNT (10^9^/L), MEDIAN (IQR)	4-10	4.60 (3.30-6.55)	4.50 (3.30-6.40)	4.60 (3.33-7.03)	0.088 (-0.005 - 0.181)	0.73
CD3+ T-CELLS (10^9^/L), MEDIAN (IQR)	0.7-2.1	0.427 (0.284-0.589)	0.447 (0.284-0.653)	0.400 (0.291-0.500)	-0.499 (-0.592 - -0.406)	0.36
CD4+ T-CELLS (10^9^/L), MEDIAN (IQR)	0.3-1.4	0.286 (0.183-0.433)	0.286 (0.195-0.466)	0.274 (0.166-0.371)	-0.505 (-0.598 - -0.412)	0.34
CD8+ T-CELLS (10^9^/L), MEDIAN (IQR)	0.2-0.9	0.131 (0.075-0.254)	0.152 (0.073-0.268)	0.101 (0.075-0.183)	-0.427 (-0.520 - -0.334)	0.34
B-CELLS (10^9^/L), MEDIAN (IQR)	0.1-0.5	0.099 (0.050-0.193)	0.091 (0.046-0.189)	0.105 (0.062-0.205)	0.290 (0.197 - 0.383)	0.39
NK-CELLS (10^9^/L), MEDIAN (IQR)	0.09-0.6	0.109 (0.075-0.156)	0.109 (0.069-0.157)	0.104 (0.086-0.154)	-0.023 (-0.116 - 0.070)	0.89
CD4+/CD8+ ratio, MEDIAN (IQR)		2.3 (1.27-3.97)	2.15 (1.28-3.97)	2.33 (1.13-4.11)	0.016 (-0.077 - 0.109)	0.93

This table shows the baseline values of lymphocyte subsets of mild versus severe COVID-19 patients and the values of the total population determined within 24 hours from ED presentation. Mild COVID-19 was defined as patients admitted to a general ward or discharged home and severe COVID-19 was defined as patients requiring ICU admission or experiencing COVID-19-related mortality. SMD, standardized mean difference; CI, confidence interval.

### 3.4 Lymphocyte subsets in COVID-19 patients with and without immunosuppressive drugs prior to COVID-19

**Figure 2:**
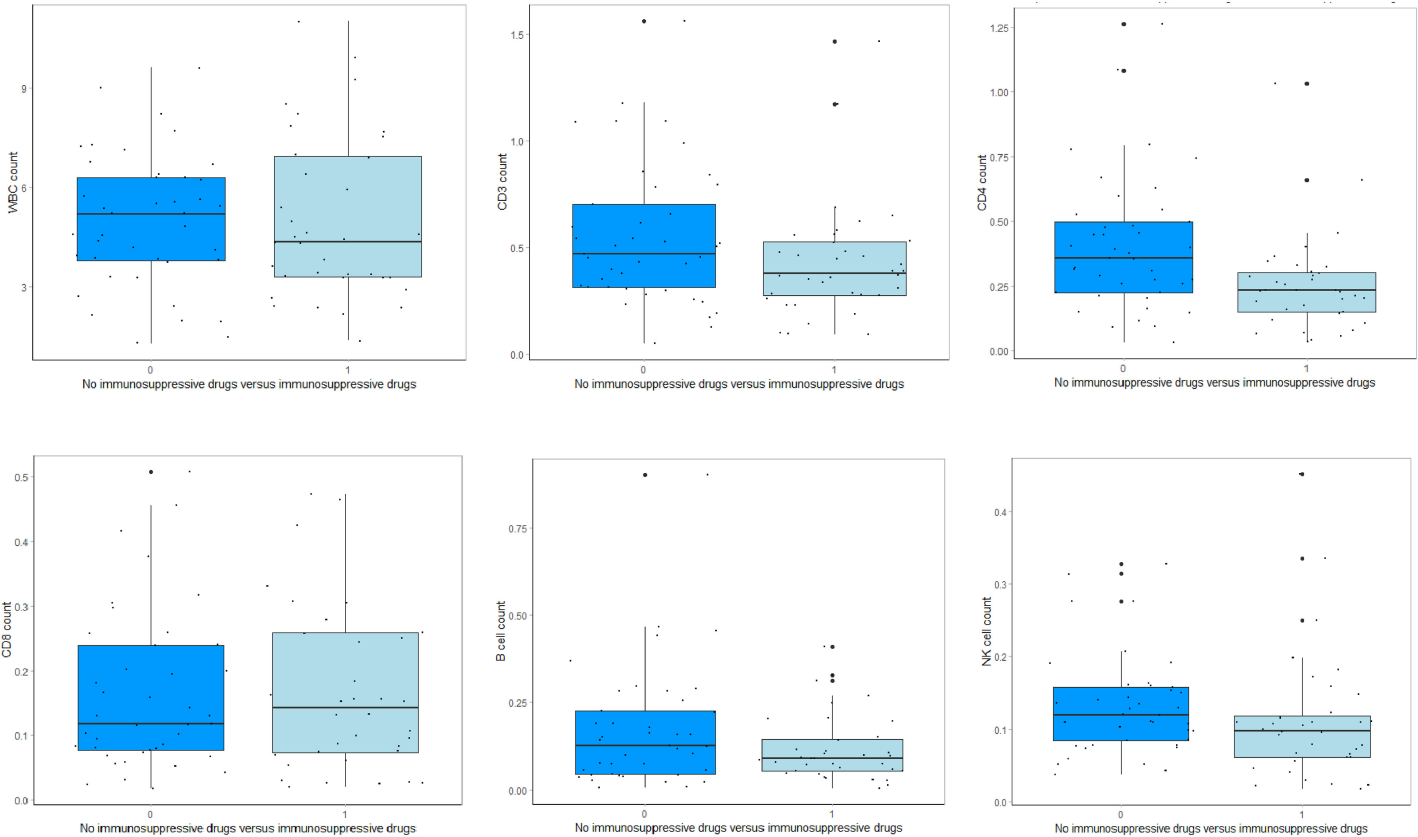
Lymphocyte subsets in patients with and without prior immunosuppressive drug use. This figure shows the lymphocyte subset values of COVID-19 patients with and without immunosuppressive drugs prior to SARS-CoV-2 infection.

We analyzed if the lymphocyte subset values were different in COVID-19 patients with and without using immunosuppressive drugs before inclusion in the study (**Supplementary Table S2**). The absolute number of CD4+ T lymphocytes was significantly lower in the group using immunosuppressive drugs (p<0.01), while no significant differences were observed for the other lymphocyte subsets (**Figure 2**). We found no significant association between the use of immunosuppressive drug and COVID-19 severity (adjusted OR 1.07, 95% CI 0.36 - 3.19, p= 0.90, **Supplementary Table S3**). Notably, 40% of the SARS-CoV-2 negative patients were using immunosuppression prior to admission, and lymphocyte subset counts were all at the bottom of or below reference values.

## 4 Discussion

No significant differences were found in lymphocyte subsets between the SARS-CoV-2 negative and SARS-CoV-2 positive patients presenting to the emergency department of our tertiary academic medical center, except for a lower CD3+ lymphocyte count in SARS-CoV-2 positive patients. In SARS-CoV-2 positive patients, patients using immunosuppressive drugs prior to COVID-19 had significantly lower CD4+ T-lymphocyte counts, but no differences in lymphocyte subsets were found between mild and severe cases. Our study found no association between lymphocyte subsets or prior immunosuppressive drug use and COVID-19 severity.

To our knowledge, CD3+ T-lymphocyte counts are not a reliable diagnostic marker to identify SARS-CoV-2 infection, but can be used as indicators of severe COVID-19 (13, 21). Previous studies, including several meta-analyses, also consistently report significantly lower counts of lymphocyte subsets CD4+ and CD8+ T-lymphocytes, NK-cells, and B-lymphocytes, along with a reduced total lymphocyte count in severely ill COVID-19 patients (12, 14). However, in our study we found no association between lymphocyte subsets and severe COVID-19. One possible explanation might be the high proportion of patients on immunosuppressive drug use prior to SARS-CoV-2 infection in our tertiary academic medical center, namely 47%. Given the dynamic nature of the host response over time, the timing of determination of lymphocytes counts, whether at ED admission, later during hospitalization, or during treatment, can be crucial, making a direct comparison with other studies challenging. Multiple previously published studies lack information on the use of immunosuppressive drugs prior to SARS-CoV-2 infection and study inclusion. Generally, immunosuppressive drug use is associated with reduced absolute lymphocytes counts (22). In our study, absolute CD4+ T-lymphocyte counts were indeed significantly lower in patients who used immunosuppressive drugs prior to SARS-CoV-2 infection. However, these lower counts were not found to be associated with severe COVID-19 outcomes. This might suggest that reduced lymphocyte subsets may only be linked to severe outcomes when caused by the infection itself. This could propose a limitation in relying solely on immunological parameters for predicting COVID-19 severity in patient populations with a high proportion of patients on immunosuppressive drugs. Alternative predictors, like organ failure-based scores like the SOFA score, may offer more reliable predictive value in settings with a high prevalence of immunosuppression (23). Interestingly, in the group of COVID-19 patients discharged home from the ED, only one patient was using immunosuppressive drugs, whereas this accounted for half of the patients in the hospitalized group. Since the use of immunosuppressive drugs prior to SARS-CoV-2 infection was not associated with severe COVID-19 in our cohort, it could be considered that the higher admission rate in this group might be attributed to the medical team’s caution rather than disease severity alone.

One strength of our study is that all patients were included within 24 hours of presentation at the ED. Additionally, the majority of our cohort had not received any COVID-19-related treatment prior to inclusion, thereby reducing the potential influence on immunological parameters at the time of sampling. Another strength of our study is the inclusion of patients with suspected COVID-19, without a confirmed diagnosis yet. This real-world approach without preselection, reflects daily clinical practice, where clinicians often face uncertainty regarding the cause of disease. A limitation of this study is the exclusion of patients admitted directly from the ED to the ICU due to logistical constraints. This exclusion potentially introduces bias, as the initially most critically ill patients have been omitted and therefore are not included in the severe COVID-19 group. However, this subset was relatively small, comprising only 17 patients, and we believe that identifying a predictive biomarker in the ED is particularly relevant for patients initially admitted to a regular ward, as these patients may benefit more from early risk stratification compared to those who already require immediate ICU care and intensive monitoring. Although this study was designed as an exploratory investigation, the limited sample size might have resulted in insufficient statistical power to detect differences within specific subgroups or to conduct sensitivity analyses. Nevertheless, we believe our findings—including the significant differences observed in CD4+ populations—are robust and contribute meaningfully to the current body of knowledge. Furthermore, in line with the FAIR data use policy, our results can be shared and utilized in future pooled analyses, enhancing their contribution to broader meta-analyses and supporting further investigation.

Our study highlights the challenges associated with using immune system-based markers for outcome prediction in a patient population characterized by a high prevalence of immunosuppressive drug use. In the future, particularly in the context of pandemic preparedness, it would be interesting to compare the predictive value of lymphocyte subsets with that of organ failure-based predictors in this patient population, or to incorporate them into a comprehensive predictive model. While measuring absolute lymphocyte subset counts provides valuable information, it does not capture the immune cell functions. Therefore, a holistic approach integrating absolute counts with assessments of immune cell functions could offer a more comprehensive understanding of immune response dynamics and improve accurate prediction in this setting (24, 25).

## 5 Conclusion

In conclusion, no significant differences were observed in lymphocyte subsets between SARS-CoV-2 negative and positive patients presenting to the emergency department of a tertiary academic medical center, except for a lower CD3+ lymphocyte count, in contrast to the majority of the previous studies. Furthermore, our study found no association between lymphocyte subsets or prior immunosuppressive drug use and COVID-19 severity among COVID-19 patients. Patients using immunosuppressive drugs prior to COVID-19 had significantly lower CD4+ T-lymphocyte counts compared to those who were not.

## Data Availability

The raw data supporting the conclusions of this article will be made available by the authors, without undue reservation.

## Supplementary material

Supplementary Table 1 Adjusted odds ratios for severe COVID-19.

Supplementary Table 2 Comparison of Lymphocyte Subsets at Admission between COVID-19 Patients with and without prior immunosuppressive drug use.

Supplementary Table 3 Multivariate analysis.
