# Supplementary material for: Exploring lymphocyte subsets in COVID-19 patients: insights from a tertiary academic medical center with a high proportion of patients on immunosuppression
Source: Front Immunol. 2024 Dec 3;15:1436637. doi: 10.3389/fimmu.2024.1436637 (PMC11649503; doi:10.3389/fimmu.2024.1436637)
Supplement: Supplementary Table 1 — Adjusted odds ratios for severe COVID-19. [file Presentation1.pdf]

Supplementary file

Supplementary Table 1. Adjusted odds ratios for severe COVID-19

	OR (95% CI)	P-VALUE
WHITE BLOOD CELL COUNT	1.044 (0.802-1.353)	0.74
CD3+ T-CELLS	0.128 (0.009-1.049)	0.19
CD4+ T-CELLS	0.088 (0.003-1.372)	0.12
CD8+ T-CELLS	0.007 (0.000-0.797)	0.18
B-CELLS	7.284 (0.180-330.946)	0.37
NK-CELLS	0.583 (0.000-460.01)	0.85

This table presents the results of multivariate logistic regression analysis adjusting for age, sex, and BMI to assess the association between lymphocyte subsets at baseline (within 24 hours from presentation to the ED) and severe COVID-19, which was defined as patients requiring ICU admission or experiencing COVID-19-related mortality. Abbreviations; NK-cells = natural killer cells, OR = odds ratio, CI = confidence interval

Supplementary Table 2. Comparison of Lymphocyte Subsets at Admission between COVID-19 Patients with and without prior immunosuppressive drug use

	NORMAL RANGE	TOTAL (N=77)	COVID-19 ANDPRIOR IMMUNOSUPPRESIVE DRUG USE (N=36)	COVID-19 NO PRIOR IMMUNOSUPPRESIVE DRUG USE (N=41)	SMD (95%CI)	P-VALUE
**WHITE BLOOD CELL COUNT (10^9/L), MEDIAN (IQR)**	4-10	4.60 (3.30-6.55)	4.35 (3.30-8.71)	5.20 (3.75-6.35)	0.097 (0.004 - 0.190)	0.69
**CD3+ T-CELLS (10^9/L), MEDIAN (IQR)**	0.7-2.1	0.427 (0.284-0.589)	0.381 (0.388-0.661)	0.457 (0.305-0.744)	-0.152 (-0.245 - -0.059)	0.14
**CD4+ T-CELLS (10^9/L), MEDIAN (IQR)**	0.3-1.4	0.286 (0.183-0.433)	0.232 (0.145-0.305)	0.376 (0.225-0.512)	-0.435 (-0.528 - -0.342)	0.001
**CD8+ T-CELLS (10^9/L), MEDIAN (IQR)**	0.2-0.9	0.131 (0.075-0.254)	0.143 (0.071-0.259)	0.130 (0.079-0.240)	0.061 (-0.032 - 0.154)	0.96
**B-CELLS (10^9/L), MEDIAN (IQR)**	0.1-0.5	0.099 (0.050-0.193)	0.090 (0.053-0.149)	0.123 (0.044-0.224)	-0.257 (-0.350 - -0.164)	0.32
**NK-CELLS (10^9/L), MEDIAN (IQR)**	0.09-0.6	0.109 (0.075-0.156)	0.098 (0.061-0.0121)	0.119 (0.081-0.161)	-0.080 (-0.173 - 0.013)	0.051

This table shows the baseline values of lymphocyte subsets of COVID-19 patients with and without immunosuppressive drugs prior to COVID-19 determined within 24 hours from ED presentation. Immunosuppressive drug use was defined as the use of systemic corticosteroids >7,5mg prednisone equivalent per day, TNF-a inhibitors, mycophenolate mofetil, calcineurin blockers, azathioprine, methotrexate, hydroxychloroquine, interleukin antagonists or others

Abbreviations; CI = confidence interval, IQR = interquartile range, SMD = standardized mean difference

Supplementary Table 3. Multivariate analysis

	OR (95% CI)	P-VALUE
**Immunosuppressive drug use**	1.074 (0.355-3.194)	0.90
**Sex**	1.825 (0.618-5.415)	
**Age**	0.993 (0.956-1.032)	
**WBC**	1.089 (0.915-1.307)	

This table presents the results of multivariate logistic regression analysis adjusting for age, sex, and white blood cell counts to assess the association between prior immunosuppressive drug use and severe or mild COVID-19

Abbreviations: WBC = white blood cell
